# A High Precision Modeling Technology of Material Surface Microtopography and Its Influence on Interface Mechanical Properties

**DOI:** 10.3390/ma14112914

**Published:** 2021-05-28

**Authors:** Yunlong Wang, Xiaokai Mu, Cong Yue, Wei Sun, Chong Liu, Qingchao Sun

**Affiliations:** 1 School of Mechanical Engineering, Dalian University of Technology, Dalian 116024, China; yunlong225@sina.com (Y.W.); suwidlut@126.com (W.S.); lichdlut@foxmail.com (C.L.); 2 Logistics Engineering College, Shanghai Maritime University, Shanghai 201306, China; yuecong234@163.com; 3 AECC Shanghai Commercial Aircraft Engine Manufacturing CO., Ltd., Shanghai 201306, China

**Keywords:** precision system, material surface, topography characteristics, rough surface simulation, mechanical properties

## Abstract

In order to accurately and effectively obtain the contact performance of the mating surface under the material surface topography characteristics, a numerical simulation method of rough surface based on the real topography characteristics and a multi-scale hierarchical algorithm of contact performance is studied in this paper. Firstly, the surface topography information of materials processed by different methods was obtained and characterized by a measuring equipment; Secondly, a non-Gaussian model considering kurtosis and skewness was established by Johnson transform based on Gaussian theory, and a rough surface digital simulation method based on real surface topography was formed; Thirdly, a multi-scale hierarchical algorithm is given to calculate the contact performance of different mating surfaces; Finally, taking the aeroengine rotor as the object, the non-Gaussian simulation method was used to simulate the mating surfaces with different topographies, and the multi-scale hierarchical algorithm was used to calculate the contact performance of different mating surfaces. Analysis results showed that the normal contact stiffness and elastic–plastic contact area between the mating surfaces of assembly 1 and assembly 2 are quite different, which further verifies the feasibility of the method. The contents of this paper allow to perform the fast and effective calculation of the mechanical properties of the mating surface, and provide a certain analysis basis for improving the surface microtopography characteristics of materials and the product performance.

## 1. Introduction

The final assembly quality and performance of a product is closely related to the quality of the material surface. In the process of machining, due to the influence of different factors such as cutting tools, equipment, and materials, damage in varying degrees will occur on the material surface. Therefore, there is always a certain micro-geometric error on the machined material surface, and there is no absolutely smooth material surface [1]. For precision machined parts, from a micro perspective, there are convex peaks and valleys of different sizes and shapes on the material surface. The influence of these different topographical features on the assembly quality of parts and the whole machine performance of products is also different [2].

The mechanical properties of assembly interface are essentially determined by the distribution of the interfacial force and the topographical characteristics of the material surface [3]. Therefore, the main way to clarify the relationship between different topographical characteristics and forces of mating surface is to find an effective method to build a contact model of material surface which conforms to real topographical characteristics and to obtain the mechanical properties of the mating surface by effective numerical analysis. This plays an important role in improving the surface topography design of a material and assembly quality of a mechanical system.

Effective simulation of material surface topography is the basis for fast and effective analysis of mating surface contact properties. At present, although some scholars have carried out relevant research on the digital simulation of material surface topography, most of them assume that the surface topography characteristics obey a certain distribution, which affects the real characteristics of material surface topography to a certain extent. Gräf et al. [4] uses fast Fourier transform (FFT) to generate rough surfaces with specified parameters. Reizer [5] used FFT and FIR methods to model rough surfaces with specified topographical characteristics. Ghosh and Sadeghi [6] constructed a rough surface model with given roughness parameters and analyzed the relationship between roughness parameters and statistical distribution parameters. Liao et al. [7] proposed an improved rough surface modeling method, which converted the solution of the autocorrelation coefficient matrix into the nonlinear least squares problem, and then constructed the rough surface satisfying the characteristics of different autocorrelation functions and statistical parameters. Mu et al. [8] studied the modeling of rough surfaces with given roughness parameters and provided the modeling method of rough surfaces with specific height distribution. Zhu et al. [9] used a computer method to effectively simulate rough surfaces with specified topographical characteristics. The simulation process mainly uses a stochastic process theory and a time series theory. Wang et al. [10] compared and analyzed the advantages and disadvantages of different rough surface simulation methods, and realized the effective simulation of three-dimensional rough surface by effective conversion between autocorrelation function and non-linear equation group. Some scholars have made contributions in topography data processing and surface quality evaluation. Goic GL et al. [11] studied a new filtering technology, which can improve the measurement accuracy by removing the artificial measurement error. Belaud V et al. [12] experimentally investigated the influence of multi-scale roughness on measurements, and proposed a model combining the Wenzel and Cassie–Baxter equations. Podsiadlo P et al. [13] used a newly developed directional blanket covering curvature method to quantify the curvature of surface topography. Bartkowiak et al. [14] studied two new methods for quantitative and visual anisotropy characterization using multiscale analysis.

In other words, the existing numerical simulation methods for material surface are all carried out under certain assumed conditions, and the simulated surface cannot accurately reflect the real situation of material surface. Therefore, it is necessary to study a material surface numerical simulation method which conforms to the true topographical characteristics, and lay a foundation for accurate acquisition of different contact properties between mating surfaces.

The coordination between parts is mainly the contact between surface micro-convex characteristics. Different micro-convex characteristics show different contact properties between mating surfaces under load [15]. Therefore, effective analysis of contact performance between mating surfaces and correlation between different topographical characteristics and contact performance will provide the basis for the subsequent design and manufacturing of mating surfaces of components. At present, the acquisition of contact performance of mating surface is mainly carried out from two aspects: construction and analysis of contact model, but all of them are based on certain assumptions. Many scholars simplify the mating surface in the process of modeling, mainly setting one of the mating surfaces as rigid plane. Jackson and Green [16] established a statistical-based elastic–plastic model using an elastic–plastic sphere in contact with a rigid plane. Jourani [17] took the contact of fractal rough surface as the contact of rough surface and rigid surface, and then carried out finite element analysis on the contact model. Thornton et al. [18] assumed the contact model between rough mating surfaces as the contact between a rigid plane and a rough surface, and analyzed the elastic and elastic–plastic problems between the mating surfaces. Pan et al. [19] constructed and analyzed contact models at different stress stages using fractal theory, and the contact characteristics of interface were analyzed by the mechanical theory, but all assumed that a rigid plane contacted with a rough surface. Kucharski and Starzynski [20] assumed that the contact model of the joint surface was composed of the contact between the rough surface with different topogical parameters and a rigid plane, and the contact performance of the mating surface was obtained by using the finite element method and the actual test. Some scholars have also studied the relationship between surface topography and friction characteristics. Bigerelle et al. [21] used the multi-scale roughness analysis method to study the wear characteristics of different particle sizes stainless steel surfaces. Lavernhe et al. [22] established an accurate prediction model for machined surface, which comprehensively considered the tool shape and the macro and micro scale characteristics of machined surface. Vulliez M et al. [23] proposed and demonstrated a method to determine the appropriate scale of multiscale parameters for certain materials and manufacturing processes to predict fatigue behavior. Goiec et al. [24] studied the relationship between the topography of different damaged rough surfaces and the processing conditions by using Gaussian filtering, wavelet transform, and the latest discrete mode decomposition. Shi R et al. [25] discussed the relationship between the surface topography parameters of rough mating surfaces and friction performance under hydrodynamic lubrication. Wang et al. [26] used the fractal contact theory to calculate the contact model of the interface composed of smooth surfaces without rough surfaces and isotropic surfaces, and obtained the contact stiffness characteristics of the micro-geometry of the contact model during the contact process.

For the construction and performance analysis of contact model for mating surface, most of the studies are based on the contact between a rigid plane and a rough surface, and the calculation process of contact performance is based on a specific topographical scale, and there is a certain deviation between the calculated results and the actual results. Therefore, based on the surface topography characteristics of actual materials, a calculation method of mating surface contact performance based on multi-scale hierarchical algorithm is studied in this paper. This provides support for analyzing the influence of topographical characteristics on the contact properties of mating surfaces at different scales.

In conclusion, the influence of different topographical characteristics on the contact properties of mating surfaces is also different. In order to quickly and accurately acquire the influence rule of material surface topography characteristics on mechanical properties of mating surface by a numerical analysis method, this paper mainly studies the digital simulation method of material surface topography characteristics. Furthermore, the influence laws of different topographical features on mechanical properties of the joint surface are obtained by a multi-scale hierarchical algorithm. Firstly, the non-Gauss theory is used to realize effective numerical simulation of material surface oriented to real surface topography information. Secondly, a multi-scale hierarchical algorithm is used to calculate the mechanical properties of mating surfaces accurately. Finally, the material surface numerical simulation method and contact performance calculation method are validated by taking the high-pressure rotor of aeroengine as an example. The content of this paper provides a basis for analyzing the influence of material topography characteristics on the contact properties of mating surfaces. It has certain guiding significance for improving surface topography design of material and matching quality of mechanical systems.

## 2. Acquisition and Characterization of Material Surface Topography

Effective measurement of material surface topography is the basis of analyzing the relationship between topography and the contact performance of mating surface [27]. In order to obtain the accurate information of material surface topography, it is necessary to measure the surface topography of machined specimens in different scales. In recent years, the measurement of material surface topography is mainly to get the two-dimensional surface profile information, which will result in the lack of information of surface topography features, and cannot truly express the real surface features of parts [28]. Therefore, the research on the technology of obtaining 3D surface topography by optical instruments will help to realize the effective expression of the real surface topography, and provide a data basis for further study of the relationship between topography characteristics and contact performance.

### 2.1. Specimens and Measuring Instrument

**Figure 1:**
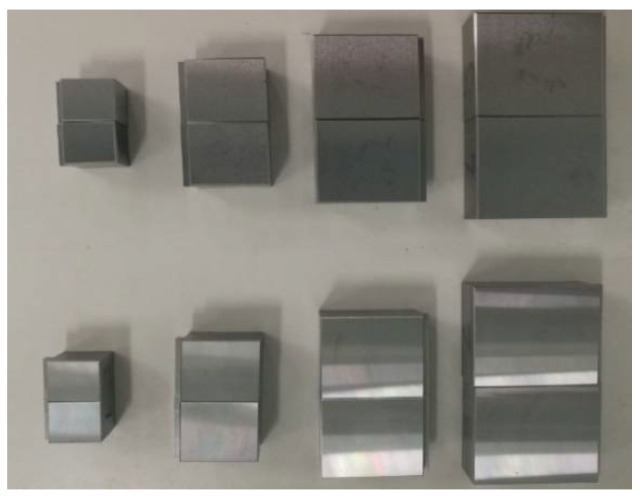
Testing Specimens.

**Figure 2:**
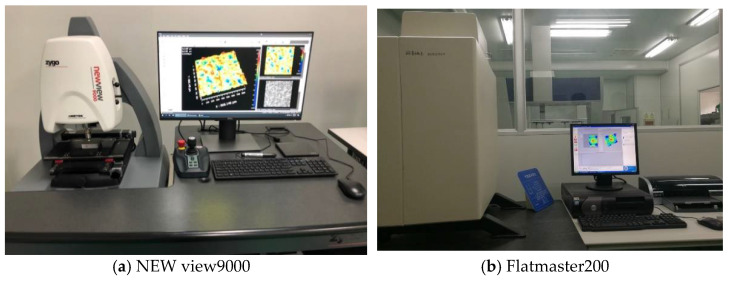
Surface topography measuring instruments.

**Figure 3:**
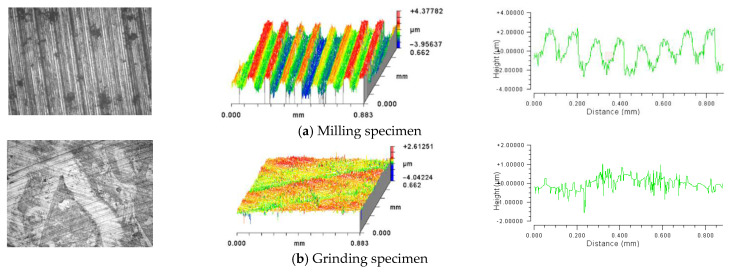
The 3D profiles and 2D roughness curves of the surface of the specimens.

In this paper, the specimens were manufactured by grinding and milling with qualified manufacturers. Multiple groups of specimens with the size of 10 mm × 10 mm\20 mm × 20 mm\30 mm × 30 mm\40 mm × 40 mm were designed and processed, respectively. Different processing methods and surface roughness requirements were adopted for each specimen and the material was 45 steel, as shown in Figure 1. The information of different topographies of the material surface is obtained by NEW view9000 with a resolution of 0.2 nm and Flatmaster200 with a resolution of 5 nm, as shown in Figure 2. The specimen surfaces were measured with 10× lens. The measured region was 0.88 × 0.66 mm and the objective lateral resolution of camera was 1.38 μm. Each surface was measured and analyzed in 5 locations, 1 in the center, and the remaining 4 in different corners. Specimen renderings of milling and grinding surfaces are illustrated in Figure 3.

### 2.2. Parametric Characterization of Surface Topography Information

The digital simulation of material surface topography is mainly based on the measured information, thus the premise and basis of numerical simulation of surface topography is the effective characterization of material surface topography information. At present, the characterization parameters of rough surface topography information mainly include the roughness average (*Ra*), root mean square roughness (*Rq*), skewness coefficient (*Rsk*), and kurtosis coefficient (*Rku*), etc. According to the standard [29], the definition and expression of several indicators are as follows:

Roughness average (*Ra*): the arithmetic average of the absolute values of the profile height deviation. As the following Equation (1):

(1) $$Ra=\left(1/L\right)\int_{0}^{L}\left|Z\left(x\right)\right|dx$$

Root mean square roughness (*Rq*): the root mean square average of the profile height deviations. As the following Equation (2):

(2) $$Rq={\left[\left(1/L\right)\int_{0}^{L}Z{\left(x\right)}^{2}dx\right]}^{1/2}$$

Skewness (*Rsk*): the asymmetry of the profile about the mean line. As the following Equation (3):

(3) $$Rsk=\frac{1}{Rq^{3}}\frac{1}{L}\int_{0}^{L}Z^{3}\left(x\right)dx$$

Kurtosis (*Rku*): the peakedness of the profile about the mean line. As the following Equation (4):

(4) $$Rku=\frac{1}{Rq^{4}}\frac{1}{L}\int_{0}^{L}Z^{4}\left(x\right)dx$$

In this paper, two kinds of surface topography measuring instruments were used to test the ten groups of specimens, and the surface topography information is expressed by different characteristic parameters. The *p*-value obtained by Shapiro Wilk test is used to judge whether the data conform to Gaussian distribution. The data information is shown in Table 1.

## 3. Numerical Simulation of Material Surface and Contact Model

The mechanical system is assembled by many different parts through different matching surfaces according to the relevant technical requirements. Therefore, the surface topography of the material plays a decisive role in the mating quality of the parts and the system performance [30]. In order to analyze the influence of different topographies on the contact performance of mating surfaces, it is necessary to obtain the contact performance of mating surfaces before assembly accurately. The most important task in the analysis process is to realize the accurate simulation of the material surface topography and the contact model of the mating surface.

**Figure 4:**
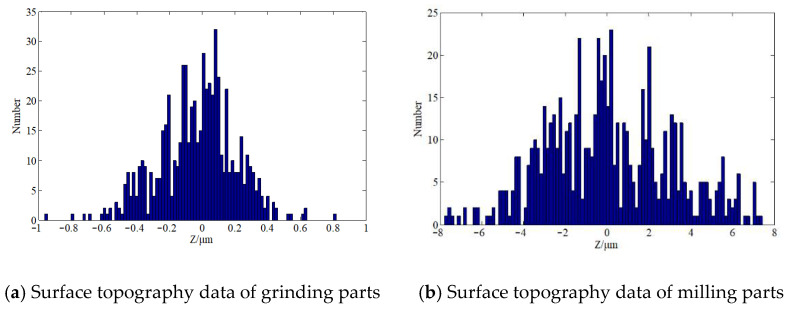
Statistical histogram of surface topography data based on the measured parts.

At present, the simulation of material surface topography is a mostly 2D profile. Although some scholars have carried out 3D surface simulation, it is basically assumed that the topography features follow the Gaussian distribution [31], which leads to the difference between the simulated surface and the actual surface, and ultimately affects the accuracy of the contact performance analysis results. In our previous research, we found that the processed topography does not completely conform to Gaussian distribution. There is a certain amount of deviation. Figure 4 shows an example of grinding and milling parts. The p-values were 0.00243 and 0.00017, respectively, after Shapiro Wilk test. In this paper, based on the Gaussian simulation method of rough surface, the skew and peak characteristics of the real topography are considered to obtain the material surface which is most suitable for the actual parts.

According to relevant research [32], the rough surface simulation method satisfying Gaussian distribution characteristics is as follows:

(1) Generating white noise sequence *η*(*m*,*n*), and the Fourier transform. As the following Equation (5):
  (5) $$A\;(k\;+\;1,l\;+\;1)\;=\;\sum_{r=0}^{m-1}\sum_{s=0}^{n-1}\eta (\;r\;+1,s\;+1)\cdot e^{\left[-2\pi i(kr/m+ls/n)\right]}$$
  where *m*,*n* represent the number of noise sequence points in x and y directions, respectively. *k* = 0, 1, 2, …, *m*−1; *l* = 0, 1, 2, …, *n*−1.
(2) The autocorrelation function is given. As the following Equation (6):
  (6) $$f\left(m,n\right)=Rq^{2}e^{\left[-2.3{\left({\left(x/L_{x}\right)}^{2}+{\left(y/L_{y}\right)}^{2}\right)}^{1/2}\right]}$$
  where *L_x_* and *L_y_* represent the correlation lengths of sequence points in x and y directions, respectively.
(3) The autocorrelation function is discretized by the discrete interval Δx and Δy, and the corresponding sequence *R*(*m*,*n*) is obtained. As the following Equation (7):
  (7) $$\left\{ \begin{array}{l} R\left(i+1,j+1\right)=f\left(i,j\right)\\ R\left(0.5m+2+i,j+1\right)=f\left(0.5m-i,j+1\right)\\ R\left(i+1,0.5n+j+2\right)=f\left(i+1,0.5n-j\right)\\ R\left(0.5m+i+2,0.5n+j+2\right)=f\left(0.5m-i,0.5n-j\right)\\ i=0,1,2\cdots \left(m/2\right)-1\\ j=0,1,2\cdots \left(n/2\right)-1 \end{array}\right.$$
(4) The corresponding power spectral density is obtained by the Fourier transform of Equation (7). As the following Equation (8):
  (8) $$P\;(k\;+\;1,l\;+\;1)\;=\;\left|\sum_{r=0}^{m-1}\sum_{s=0}^{n-1}R(\;r\;+1,s\;+1)\cdot e^{\left[-2\pi i(kr/m+ls/n)\right]}\right|$$
(5) For the power spectral density of white noise, *C* = 1, thus the transfer function *H* is shown in Equation (9):
  (9) $$H\;(m,n)\;=\;\frac{P^{1/2}}{C^{1/2}}=P^{1/2}$$
(6) The height sequence is obtained by inverse Fourier transform of point multiplication (*A*·*H*). As the following Equation (10):
  (10) $$z_{0}\;(k\;+\;1,l\;+\;1)\;=\;\frac{1}{mn}\sum_{r=0}^{m-1}\sum_{s=0}^{n-1}(\;A\cdot H)\cdot e^{\left[2\pi i(kr/m+ls/n)\right]}$$
(7) The Gaussian surface sequence *Z*(*m*,*n*) is obtained by filtering the Equation (10).

On the basis of obtaining a Gaussian distribution sequence, in order to generate a non- Gaussian sequence which is more consistent with the actual topography characteristics, it is necessary to carry out Johnson transformation on the Gaussian distribution sequence. The purpose is to consider the skewness and kurtosis of the actual surface topography, so as to make the simulated topography closer to the actual situation.

According to previous research [33], this paper mainly uses the logarithmic normal system in the Johnson transformation system, as is shown in the Equation (11):

(11) $$z_{1}\;=\gamma +\delta \log\left(\frac{z_{2}-\xi }{\lambda }\right),\;\left(z_{2}>\xi \right)$$

here, *z*_1_ is a Gaussian random sequence, *z*_2_ is a non-Gaussian random sequence, and *γ*, *δ*, *ξ*, and *λ* are the constants in the conversion process.

By transforming Equation (11), we obtain Equation (12):

(12) $$z_{2}=\xi +\lambda \cdot e^{\frac{z_{1}-\gamma }{\delta }},\;\left(z_{2}>\xi \right)$$

Assuming that *Rsk*_1_ and *Rku*_1_ are the input skewness and kurtosis of the non-Gaussian sequence, the *Rsk_z_* and *Rku_z_* of the output sequence can be obtained by Equations (13) and (14).

(13) $$Rsk_{z}=\frac{\sum_{k=0}^{N-1}\sum_{l=0}^{M-1}h{\left(k,l\right)}^{3}}{{\left(\sum_{k=0}^{N-1}\sum_{l=0}^{M-1}h{\left(k,l\right)}^{2}\right)}^{3/2}}Rsk_{1}$$

(14) $$Rku_{z}=\frac{\sum_{k=0}^{N-1}\sum_{l=0}^{M-1}h{\left(k,l\right)}^{4}Rku_{1}+6\sum_{k=0}^{N-1}\sum_{p=k+1}^{N}\sum_{l=0}^{M-1}\sum_{q=l+1}^{M}h{\left(k,l\right)}^{2}h{\left(p,q\right)}^{2}}{{\left(\sum_{k=0}^{N-1}\sum_{l=0}^{M-1}h{\left(k,l\right)}^{2}\right)}^{2}}$$

Then, the non-Gaussian distribution sequence including skewness and kurtosis is obtained by filtering *z*_2_.

**Figure 5:**
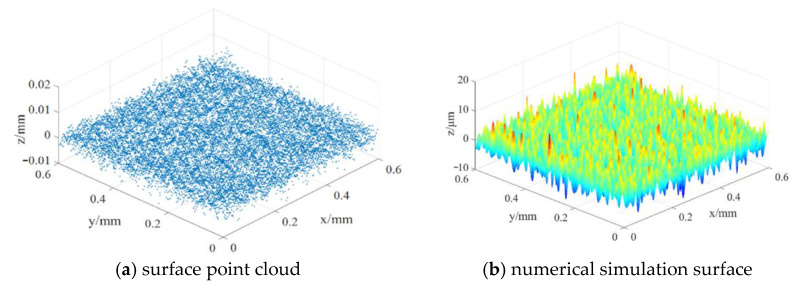
Non-Gaussian simulation of material surface.

According to the surface topography information of the measured material, the numerical simulation of specimen 2 (*Rq* = 0.40 μm, *Rsk* = −0.41, *Rku* = 3.43, *p*-value = 0.00243) is carried out in this part, as shown in Figure 5.

To obtain the numerical simulation surface which can meet the material topography characteristics is the premise of building the contact model of parts. In this paper, the contact model of parts is obtained by reverse reconstruction of the simulated surface which conforms to the topography characteristics of the real material surface, so as to realize the digitization of the contact model, which will lay the foundation for the rapid analysis of the contact performance of the mating surface under different topographies.

**Figure 6:**
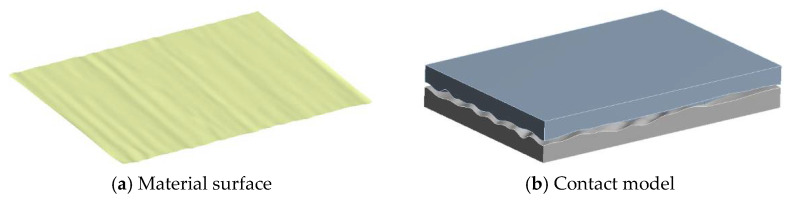
Construct of the contact model.

In the process of reverse modeling, the digital shape editing (DSE) module of 3D software is mainly used to import and process the point cloud data; the fast surface reconstruction (QSR) module is used to fit the processed point cloud data; finally, the creative shape design (GSD) module is used to carry out a series of operations on the surface model to obtain the final part surface model and mating surface contact model. Figure 6 shows the contact model of mating surface generated by reverse modeling.

## 4. Multi-Scale Hierarchical Algorithm of Mating Surfaces’ Contact Performance

The material surface is composed of many different micro-convex bodies, and the fit of two parts is actually the contact between different material surfaces. Due to the existence of different asperities on the surface, the matching between parts is actually caused by the mutual contact between some asperities, thus the influence of micro-convex shape on the contact performance of different materials will be different [34]. In order to obtain the most accurate contact performance between the mating surfaces, a multi-scale hierarchical algorithm for contact performance based on macro and micro surface topography is proposed in this paper, which is considered as different micro-convex on the material surface.

According to the mechanical theory, there is a certain functional relationship between the contact stiffness and the contact pressure of mating surface. Assuming that the contact stiffness of a small contact area (in micro scale) under the influence of the contact pressure $p_{n}^{\left(i\right)}$ is $K_{n}^{\left(i\right)}$, according to the Yoshimura integral method [35]. As the following Equation (15):

(15) $$K_{n}^{\left(i+1\right)}=\iint_{S_{}^{\left(i+1\right)}}K_{n}^{\left(i\right)}(p_{n}{}^{\left(i+1\right)})dS$$

$K_{n}^{\left(i+1\right)}$ and $p_{n}^{\left(i+1\right)}$ represent the contact stiffness and the contact pressure in the larger contact area (macro-scale) respectively.

For the contact stiffness between mating surface, the micro-scale contact stiffness will provide local stiffness information for the calculation of macro-scale contact performance, and the calculation of macro-scale contact performance will also provide the necessary contact pressure for the acquisition of micro-scale contact stiffness. The contact stiffness of the whole mating surface can be obtained by the mutual progression of macro and micro scales. As the following Equation (16):

(16) $$K_{nS}=\iint_{S}k_{n}(p_{n})dS$$

For the micro scale, the contact pressure and deformation of each contact point are mainly obtained by using the finite element method to analyze the contact performance of the mating surface, and the power exponent form is used to fit it [36,37,38]. As the following Equation (17):

(17) $$\delta_{n}=cp_{n}{}^{m}$$

It is equivalent to the Equation (18):

(18) $$k_{n}=\alpha p_{n}{}^{\beta }$$

where $=\frac{1}{\mathrm{cm}}$, β=1−m.

For the macro scale, the total contact stiffness of the mating surface can be obtained by discretizing Equation (19),

(19) $$K_{n}=\frac{\Delta x\Delta y}{S_{0}}\sum_{i=1}^{M}\sum_{j=1}^{N}k_{nij}(p_{nij})$$

where Δ*x* and Δ*y* are the coordinate intervals between two adjacent contact points in *x* and *y* directions, respectively; *k_nij_* is the local contact stiffness obtained under the micro-scale feature; *p_nij_* is the contact pressure of each node under the micro-scale feature; *M* and *N* are the total number of contact nodes in *x* and *y* directions, respectively; and *S*_0_ is the nominal contact area at the mating surface.

## 5. Example

### 5.1. Introduction

The rotor is assembled by several disk stages. Because its working environment has the characteristics of high temperature, high pressure, and high speed, it has high requirements for assembly quality. At the same time, due to its complex structure, it will take a lot of time to correct the out of tolerance problem in the assembly process, which greatly increases the cost. Through an accurate assembly simulation, it has important implications to control the assembly quality very well.

**Figure 7:**
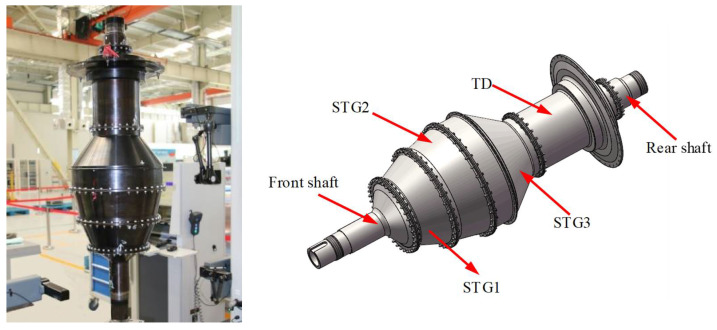
The rotor system of an aeroengine.

In this paper, we took the assembly of 3rd stage (STG3) and turbine disc (TD) for aero-engine high-pressure rotor as the object. The end face errors measured on the mating surface of the 3rd stage disk and turbine disk are counted respectively, and then the numerical simulation method is used to realize the numerical simulation of the mating surface and contact model of the assembly. Figure 7 shows the rotor system of an aeroengine.

### 5.2. Numerical Simulation of Mating Face and Contact Model

**Figure 8:**
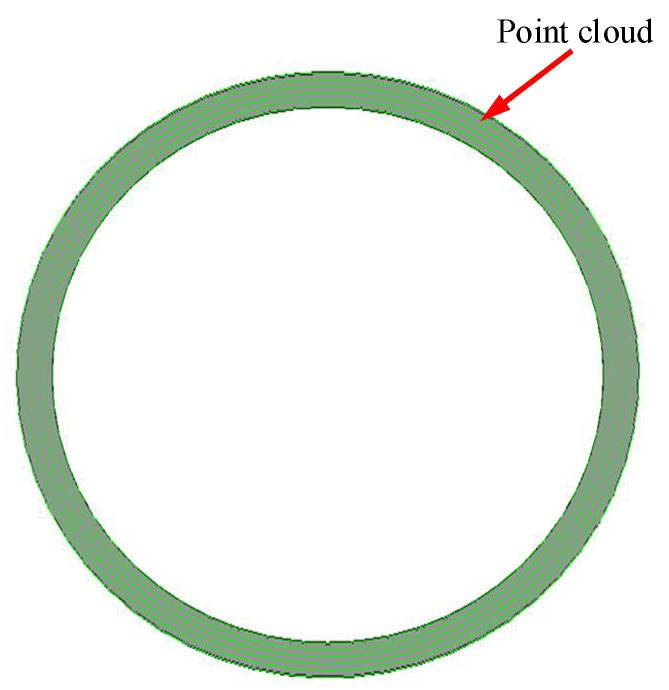
Numerical simulation of mating face.

For the upper end surface of the STG3, the statistics and characterization of the topography showed that *Rq* = 0.20 μm, *Rsk* = −0.02, and *Rku* = 2.02. For the lower end surface of TD: *R_q_* = 0.80 μm, *Rsk* = 0.50, and *Rku* = 1.92; then, the numerical simulation surface based on the measured data characteristics is obtained by using the non-Gaussian surface simulation method. Figure 8 shows the point cloud data and surface model of STG3 upper end face generated by the non-Gaussian simulation method. The same method is used to generate the lower end surface of TD.

**Figure 9:**
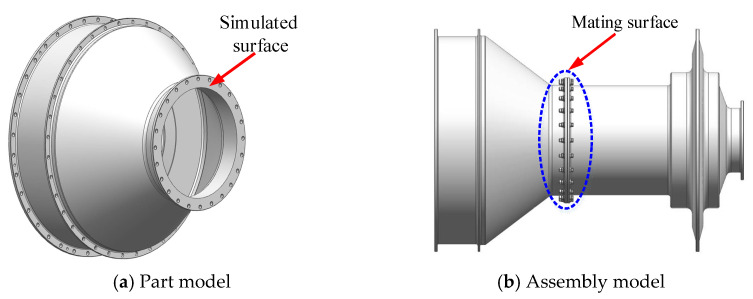
Reverse reconstruction of contact model for rotor system.

Based on the matching end surface model, the part model and assembly contact model which is made up of STG3 and TD are constructed by reverse reconstruction technology, as shown in Figure 9.

It can be seen from Figure 9 that the 3D surface model and assembly model can be generated by using the surface numerical simulation method and reverse reconstruction technology, which can provide an effective model basis for the subsequent analysis of the contact performance of the mating surface.

### 5.3. Contact Performances Analysis of Mating Surfaces

In order to explain the influence of different topographical characteristics on the contact performance of mechanical structures more intuitively, the assembly of STG3 and TD is taken as the object, and uses the finite element method to obtain the contact performance of the mating surface. According to the different errors of the upper end face of the STG3, the assembly contact model satisfying the error distribution of different end faces was generated by using the reverse reconstruction method, and the contact analysis was carried out respectively.

**Figure 10:**
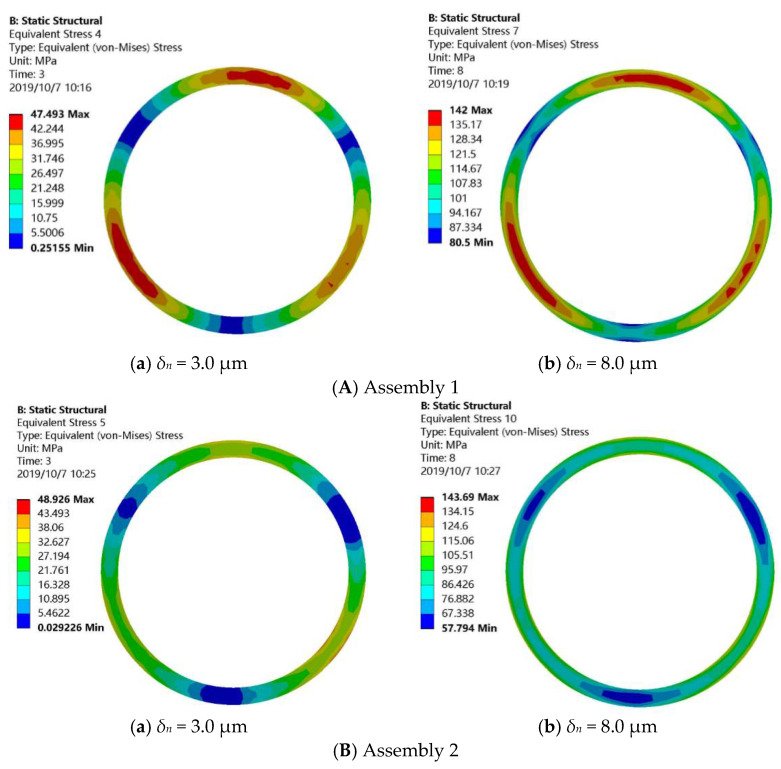
Contact stress nephogram of mating surface for different assemblies.

Figure 10 shows the contact stress at the mating surface of the assembly under different displacements. Figure 10A shows the assembly analysis results of the STG3 and TD constructed in the previous section (assembly 1). Figure 10B shows the assembly analysis results of the lower end face of the turbine disk and the upper end face (*Rq* = 0.22 μm, *Rsk* = −0.09, *Rku* = 1.92) of the third stage disk (assembly 2).

As can be seen from Figure 10, the contact stress at the mating surface increases with the increase of compression displacement due to the existence of mating surface error, and the contact area is concentrated in a few small areas. In addition, for Figure 10A,B, under the same displacement, the influence of different end surface errors on the contact performance of the mating surface is also different, which indicates that the surface topography of the material has a certain influence on the contact performance of the mating surface.

### 5.4. Results and Discussion

In order to illustrate the influence of different end surface errors on the contact performance of mating surfaces, the contact stiffness and contact area were analyzed respectively.

(a) Contact stiffness analysis

**Figure 11:**
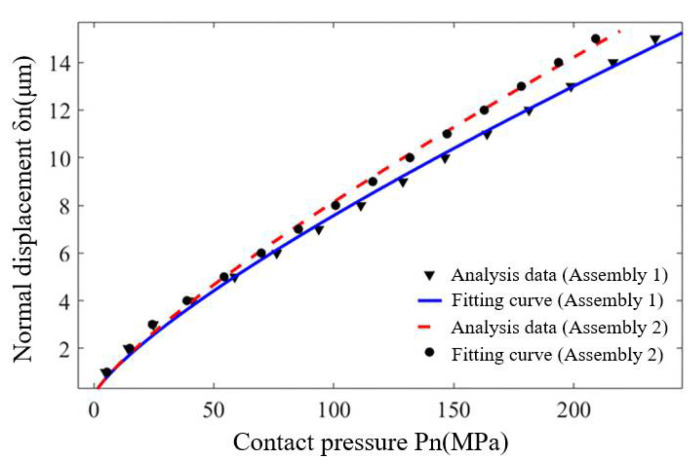
Fitting curves of mating surface contact performance of different assemblies.

The contact performances of the two assemblies were calculated by using the multi-scale hierarchical algorithm of contact performance, and the average contact pressure *p_n_* and corresponding normal displacement *δ*_n_ between the mating surfaces were extracted respectively, which were fitted by Equation (17), as shown in Figure 11.

It can be seen from the average contact pressure of assembly 1 and assembly 2 at the mating surface are 112.95 Mpa and 102.16 Mpa, respectively, and the relative error is 9.55%. In addition, the undetermined parameters *c* and *m* of different fitting curves can be obtained according to Equation (17), from which the required *α* and *β* values in Equation (18) can be calculated, as shown in Table 2.

From Table 2 and Equation (18), the relationship between normal contact stiffness *k_n_* and contact pressure *p_n_* of different assemblies can be obtained as follows:

For assembly 1, the normal contact stiffness of the mating surface is expressed as the following Equation (20):

(20) $$k_{n}=6.1366p_{n}{}^{0.2203}$$

Similarly, the normal contact stiffness of assembly 2 is shown in Equation (21).

(21) $$k_{n}=6.1957p_{n}{}^{0.1958}$$

According to Equations (20) and (21), the normal contact stiffness of assembly 1 and assembly 2 is 17.39 MPa/μm and 15.33 MPa/μm, respectively, and the relative error is 11.85%.

(b) Contact area analysis

**Figure 12:**
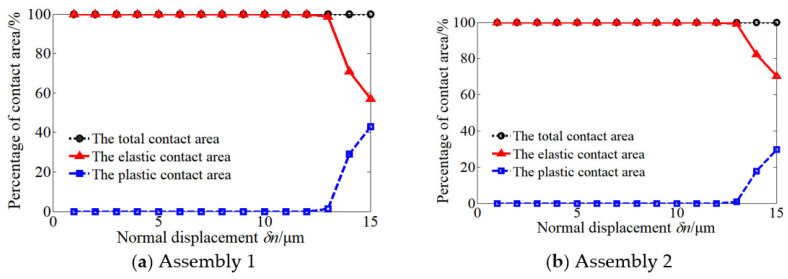
Relationship between normal displacement and contact area for different assemblies.

In order to further explain the influence of surface topography on the contact performance of the assembly, the contact areas of different assemblies under load were analyzed. Figure 12 shows the displacement–contact area percentage relation curves of different assemblies.

It can be seen from Figure 12 that the elastic deformation mainly exists at the mating surface when the applied displacement between the mating surfaces is less than 13 μm for assembly 1 and assembly 2.

When the displacement is greater than 13 μm, the percentage of elastic contact area decreases to 58% and the percentage of plastic contact area increases to 42% for assembly 1 with a small end surface error, while for assembly 2, the percentage of elastic contact area is 70% and the percentage of plastic contact area is 30%.

A fast calculation method of contact performance of mating surface based on measured data was studied. The contact stiffness and contact area obtained by the calculation verify the influence of different topographies on the contact performance of the mating surface, which further proves the feasibility of the calculation method.

In this paper, the multi-scale hierarchical algorithm was used to calculate the contact performance of the mating surface with different topographies, and the error of the contact stiffness of the mating surface with two topographies was 11.85%. In addition, when the small displacement load is applied between the two surfaces, there is mainly elastic deformation between the two surfaces, and there is basically no plastic deformation. However, when a large displacement load is applied between the mating surfaces, the percentage of elastic contact area decreases and the percentage of plastic contact area increases for the assembly with small manufacturing error at the mating surface. Similarly, for the assembly with large manufacturing error at the mating surface, the situation is just the opposite: the elastic contact area between the mating surfaces accounts for the majority, and the plastic contact area only accounts for a small part. Two different assemblies show different contact stiffness and contact area under the same displacement: under the condition of early displacement load, the existence of surface topography will cause more initial micro convex bodies to be crushed, which makes the plastic deformation area between mating surfaces take up a larger proportion. With the gradual increase of load, the micro convex bodies between mating surfaces will contact more fully, which will lead to greater contact stiffness and plastic contact area between mating surfaces.

There are still some deficiencies in this study. As an example, aeroengine rotor parts have little difference in surface topography parameters, and the structure of mating surface is complex; thus, it is difficult to carry out experimental verification. In order to further clarify the relationship between topography parameters and mechanical property parameters, mechanical experiments should be added in the follow-up research.

## 6. Conclusions

The effective construction of material surface model and contact model by numerical simulation method is the premise and foundation to obtain the contact performance of mating surface. This study mainly presents a numerical simulation method of surface topography based on the real topography characteristics of materials, and obtained different mechanical properties of the mating surface through the multi-scale hierarchical algorithm of contact performance; then, it analyzed the influence of different surface topography characteristics on the contact performance of the mating surface. The conclusions are as follows:

(1) A non-Gaussian theoretical simulation method of material surface oriented to real surface topography is proposed, which realized the digital simulation of different material surfaces and provided a model basis for analyzing the contact properties of mating surfaces.

(2) A multi-scale hierarchical algorithm for the contact performance of mating surfaces is proposed, which can effectively calculate the different contact performance of mating surfaces, and provide a basis for analyzing the influence of different topographies on the contact performance of mating surfaces.

(3) Taking high-pressure rotor of aeroengine as an example, a non-Gaussian simulation method and a multi-scale hierarchical algorithm are used to obtain the contact performance of mating surfaces, and the influence of different topographical characteristics on the contact performance of mating surfaces was analyzed.

The content of this study allows to quickly realize the effective calculation of the contact performance of the matching surface, which provides a certain analysis basis for improving the surface topography characteristics of materials and the matching performance of products, and has important engineering reference value for effectively predicting the contact performance of mechanical systems.

## Figures and Tables

**Table 1:** Characterization of surface topography parameters of specimens.

Number	*Ra* (μm)	*Rq* (μm)	*Rsk*	*Rku*	*p*-Value	Processing Method
1	0.05	0.06	0.02	4.67	0.00492	grinding
2	0.31	0.40	−0.41	3.43	0.00243	grinding
3	0.54	0.67	−0.12	3.48	0.00169	grinding
4	1.53	1.99	−0.02	2.49	0.00115	grinding
5	1.68	2.15	0.12	3.14	0.00121	grinding
6	0.15	0.21	−0.03	5.37	3.712 × 10^−4^	milling
7	0.24	0.31	−0.52	4.57	1.713 × 10^−4^	milling
8	0.92	1.13	−0.38	2.15	3.137 × 10^−6^	milling
9	0.98	1.22	0.19	2.81	1.085 × 10^−7^	milling
10	1.52	1.84	−0.04	2.11	6.759 × 10^−8^	milling

**Table 2:** The undetermined coefficients of different assemblies.

Coefficients	*c*	*m*	*α*	*β*
Assembly 1	0.2090	0.7797	6.1366	0.2203
Assembly 2	0.2007	0.8042	6.1957	0.1958

## Data Availability

Not applicable.
